# Chromosome structural variation analysis reveals lung cancer-associated gene regulatory networks in rheumatoid arthritis patients

**DOI:** 10.1186/s12920-025-02273-7

**Published:** 2025-12-23

**Authors:** Heng Li, Liping Ding, Rui Liao, Nini Li, Xiaoping Hong, Zhenyou Jiang, Dongzhou Liu

**Affiliations:** 1 Department of Rheumatology and Immunology, The Second Clinical Medical College, Jinan University (Shenzhen People’s Hospital), Shenzhen, 518020 China; 2 Integrated Chinese and Western Medicine Postdoctoral research station, Jinan University, Guangzhou, 510632 China; 3 Department of Pathology, The Second Clinical Medical College, Jinan University (Shenzhen People’s Hospital), Shenzhen, 518020 China; 4 Department of Microbiology and Immunology, School of Medicine, Jinan University, Guangzhou, China

**Keywords:** Chromosome structural variations, Lung cancer, Rheumatoid arthritis, Interstitial lung disease

## Abstract

**Background:**

Chromosomal structural variations (CSVs) that comprise multiple gene mutations are important determinants for multiple diseases. However, the relationship between CSVs, rheumatoid arthritis (RA), and lung cancer is not well understood.

**Materials and methods:**

In this study, we analyzed CSV associations and differences between RA and RA with lung cancer (RA LC) using genome sequencing, with RA-associated interstitial lung disease (RA ILD) as a disease control. First, we analyzed the CSVs of each individual. Then, we identified common CSVs within each disease group and finally analyzed specific CSVs between different diseases. Gene Ontology/KEGG terms, canonical pathways, and feature gene sets were used for the functional annotation and analysis of CSV-related pathways.

**Results:**

Cell size regulation and axon guidance were mutated in all disease groups. Protein deubiquitination was mutated in RA LC, while the negative regulation of extractable stroma and protein catabolism was mutated in RA ILD. Characterization of clinical data also revealed correlations with these specific pathways.

**Conclusion:**

This study identifies common and specific CSVs and associated pathways for RA, LC, and ILD, uncovering key genetic factors that provide new insights into their diagnosis and treatment.

**Supplementary Information:**

The online version contains supplementary material available at 10.1186/s12920-025-02273-7.

## Introduction

Compared to the general population, patients treated with biologic therapies have an increased risk of developing lung cancer, with a standardized incidence ratio (SIR) of 1.35 [1.14; 1.60] [1]. Connective tissue diseases contribute to a poorer prognosis in lung cancer patients, particularly in those with interstitial lung disease (ILD) and Qi deficiency, both of which are associated with reduced survival in some cases [2]. Rheumatoid arthritis (RA) is a systemic inflammatory autoimmune disease with a global incidence of ~ 0.5–1.0.5.0% [3–5]. The main features are persistent synovitis, systemic inflammation, and the presence of autoantibodies, which ultimately lead to joint injury, disability, and complications of various systems [6, 7]. Smoking prevalence in patients with rheumatoid arthritis (RA) exceeds that reported in the general population. In addition, RA patients who smoked were more likely to develop lung cancer than smokers without RA [8]. In 2016, Curtis et al. found that among 5671 patients treated with tropinib, the most common malignant tumor was lung cancer (*n* = 24, 0.42%) [9]. In 2022, Chatzidionysiou et al. found 590 cases of lung cancer in 44,101 RA patients (56/100000), higher than the general population (HR 1.76, 95% CI 1.60 to 1.93); This increased risk remained after adjustment for smoking [10]. Although the mechanisms underlying this phenomenon are not yet fully understood, genetic factors appear to play a critical role [11].

Genetic factors contribute to 60% of RA susceptibility [12, 13]. Researchers found that more than 100 loci are associated with RA, of which 80% of the risk variants are located in non-coding regions, affecting gene expression and mRNA stability [14]. The HLA-II gene is associated with RA susceptibility (accounts for 30%) [15]. Inherited mutations in HLA-DRB1 and IRX1 were shown to contribute to the pathogenesis of RA [16, 17], and polymorphisms in other classical HLA genes such as HLA-DPB1, HLA-B, and HLA-A genes also increased RA susceptibility [18]. Non-classical HLA gene synonymous mutations in HLA-DOA are an independent risk of anti-citrullin protein autoantibodies (ACPA)-positive RA [19]. In addition, heterogeneous single nucleotide polymorphisms in genes encoding protein tyrosine phosphatase (PTPN22) outside the HLA region were associated with RA [20]. A functional mutant (R620W) of PTPN22 widely expressed in hematopoietic cells has been shown to cause a two-fold increased risk of RA and other autoimmune diseases [21]. Xu Huji et al. firstly identified MMEL1 and CTLA4 as susceptibility genes for RA in Han Chinese [22, 23]. The further international collaborative study uncovered 101 RA pathogenic loci, which shed light on developing new drugs [24]. A genome-wide association of meta-analysis revealed that RA-associated single nucleotide polymorphisms (SNPs) show significant enrichment in the enhancer regions of T cells and NK cells [25]. A study based on the Wellcome Trust Case Control Consortium (WTCCC) SNP datasets identified 49 SNPs presumed to be associated with RA [26]. In addition to HLA-DRB1 and PTPN22, TRAF1-C5 and TAT4 have also been linked to RA susceptibility [27–29]. These studies demonstrate that genetic variation is closely related to the pathogenesis of RA.

Previous studies on RA have primarily focused on mutations in individual genes or amino acids, without giving much attention to chromosomal variations. However, CSVs involve many genes and are crucial to genetic variation. Therefore, it is of significant importance to analyze CSVs the genome-wide level in RA patients, as this can help improve our understanding of the pathogenic mechanisms.

## Materials and methods

### Patients and controls

Patients and normal controls were recruited from the Department of Rheumatology, Shenzhen People’s Hospital, the second clinical medical college, Jinan University, China, from January 2020 to March 2022. All participants were over 18 years old and provided written informed consent. Since RA patients are primarily female (80%), we mainly enrolled female patients (90%) in this study. Patients with RA strictly fulfilled the 2010 ACR/EULAR classification criteria, and their medical histories and biochemical test results were obtained from the hospital information system. Peripheral blood mononuclear cells (PBMCs) were stored at −80℃ in a biospecimen bank. This study was approved by the ethics committee of Shenzhen People’s Hospital, the second clinical medical college, Jinan University, China, and was conducted in accordance with the Declaration of Helsinki. Informed consent was obtained from all subjects and/or their legal guardian(s).

### Whole-genome sequencing (WGS) and quality control (QC)

The genome sequencing was conducted by BGI (BGI Genomics Co., Ltd. Shenzhen, China) using the DNBSEQ platform for high-throughput sequencing. This platform ensures that each sample meets the required data output standards. Quality control (QC) for the samples involved multiple steps. Initially, DNA samples were fragmented using either a Covaris ultrasonication system or a fragmentation enzyme to produce DNA fragments of approximately 350 bp. These fragments then underwent end repair, addition of an “A” base to the 3’ ends, and ligation of sequencing adapters. For some libraries, linear amplification (LM-PCR) was performed, although this step was omitted for PCR-free libraries. After amplification or adapter ligation, the products were subjected to single-strand separation and circularization, with the circularized libraries undergoing rolling circle amplification (RCA) to produce DNA Nano Balls (DNBs). The DNBs were then quality-controlled, and upon passing QC, they were sequenced on the DNBSEQ platform. The sequencing generated paired-end reads stored in FASTQ format as raw data. On average, each sample produced 100,471.76 Gb of raw bases. After removing low-quality reads, each sample yielded an average of 658,872,994 clean reads (98,830.95 Gb), with high sequencing quality indicated by Q20 = 96.87% and Q30 = 92.36%. The average GC content was 40.90%.

### Identification of chromosomal structural variations (CSV)

CSVs were detected using Breakdancer software with default parameter settings. This method primarily relies on the distances between paired-end reads and their alignment directions, utilizing an algorithm based on discordant read pairs to identify structural variations. The specific command used for detecting structural variations was: breakdancer_max sample.cfg > sample.out.

### Functional annotation and pathway analysis

DAVID Bioinformatics Resources 6.8 (https://david.ncifcrf.gov/) was employed for functional annotation of the key differential genes primarily associated with either RA or lung cancer [30]. Pathways were enriched using web-based knowledge databases KEGG, GO and BioCarta [31]. KEGG pathway mapping was utilized to generate signaling pathways.

### Protein-protein interaction (PPI) analysis

In order to investigate the interactions between proteins, we established a PPI network in the STRING database (Version 11.0; http://string-db.org/). The analysis involves identifying protein-protein interactions, determining their binding strengths, and characterizing the resulting protein complexes.

### Clinical data

Safety assessment was performed by blood routine examination, liver and kidney function and immunoglobulin. Serological markers included rheumatoid factor (RF), C-reactive protein (CRP), erythrocyte sedimentation rate (ESR), anti-CCP antibodies, complement C4 (C4), neutrophil and lymphocyte.

### Statistical analysis

Statistical analysis was conducted using SPSS 17.0 software (SPSS, Chicago, USA). Violin plots and box plots were used to visualize the distribution of key variables across different disease groups and were generated using GraphPad Prism 5. The measurement data were expressed as “r” with a 95% confidence interval (CI). Normally distributed data were compared using ANOVA or t-tests, while nonparametric methods were applied to data that did not follow a normal distribution. Data were presented as mean ± standard deviation (SD). The choice of statistical method was determined according to the number of samples. A Bonferroni correction was applied to adjust for multiple testing, and a p-value of < 0.05 was considered statistically significant.

## Results

### Determine the chromosomal structural variation of the individual patient

The patients in this study were divided into three groups: primary rheumatoid arthritis (RA), rheumatoid arthritis with lung cancer (RA LC) and rheumatoid arthritis with interstitial lung (RA ILD), the human reference genome (GRCh38/HG38) was used as a control to identify the differences in CSV between different groups. The analysis flow is briefly as follows: each individual was first analyzed for CSVs, then intersected to obtain the common CSVs in each group, and finally to determine the specific differences between different groups. The inactivated genes in CSVs were subsequently analyzed. The signaling pathways associated with various diseases were finally identified through enrichment analysis of the critical genes.

**Fig. 1:**
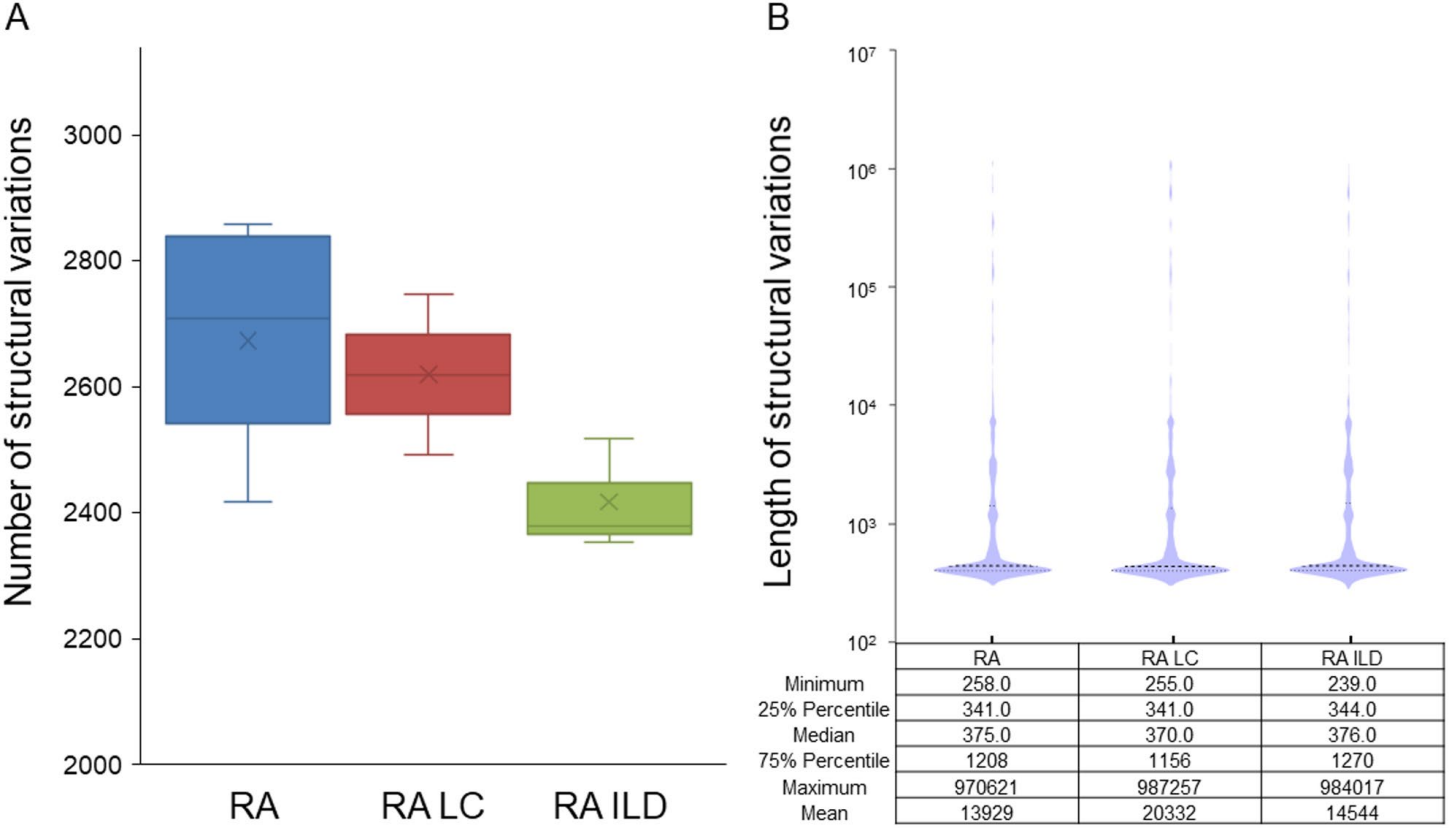
Number (**A**) and length (**B**) of CSVs for different diseases. Descriptive statistics (minimum, 25th percentile, median, 75th percentile, maximum, and mean) for each group are displayed below the violin plots

Deletion mutation was the primary type of chromosomal structural variation of RA, RA LC, and RA ILD. The number of structural variants in the three diseases was similar; the median of RA ILD is the most miniature, which is 88.1% of RA (Fig. 1A)。 The length characteristics in the three groups were consistent, with similar median and quartiles (Fig. 1B). Among them, the structural variation of RA LC has the most extended average length, and RA is the shortest.

### Exclude individual differences to determine the chromosomal variation for each disease

**Fig. 2:**
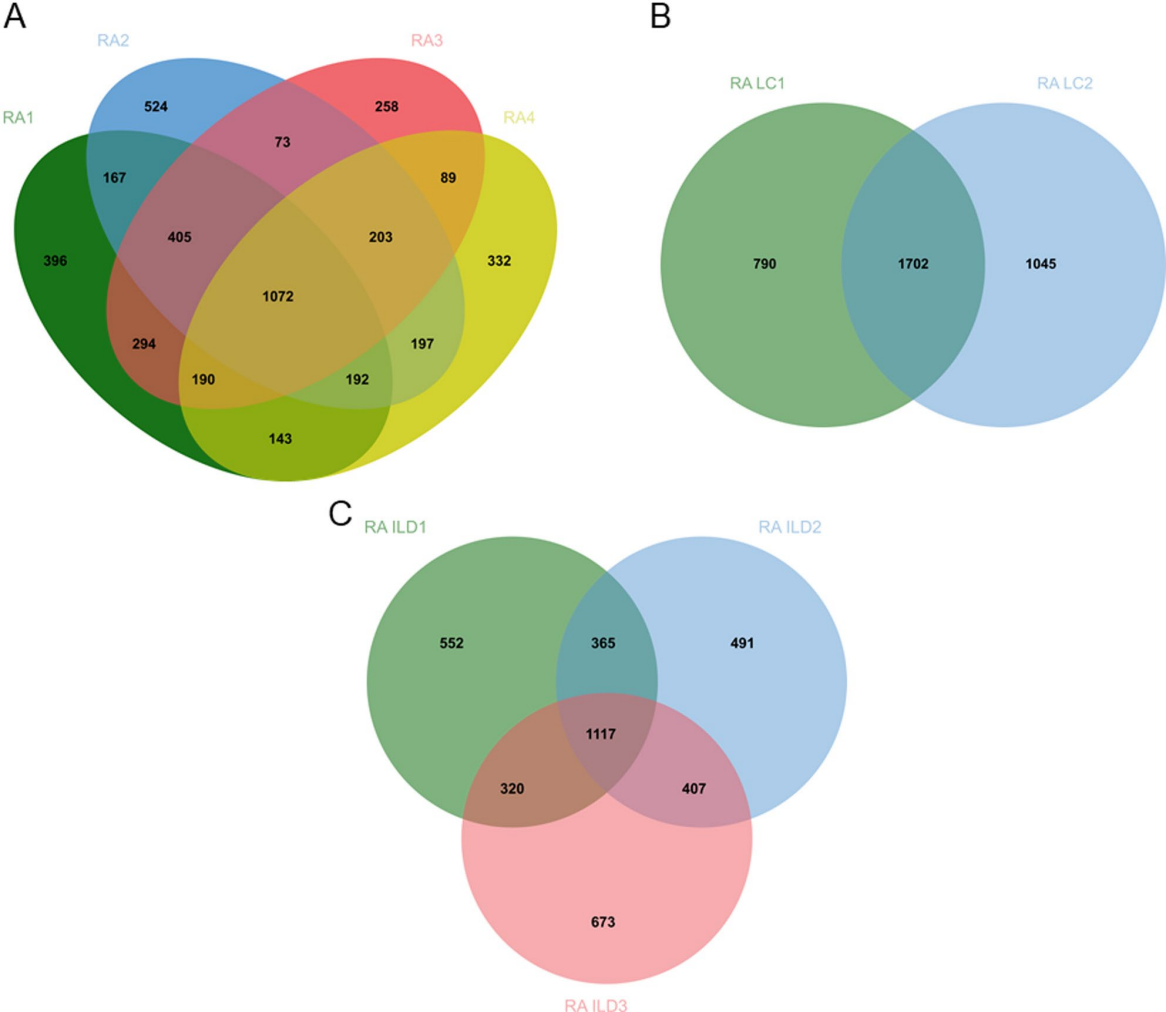
Chromosomal structural variations for three disease groups (*n* = 9). Venn diagrams illustrate the overlap and distinct chromosomal structural variations identified in three disease groups: **A** Rheumatoid Arthritis (RA), **B** Rheumatoid Arthritis with Lung Cancer (RA LC), and **C** Rheumatoid Arthritis with Interstitial Lung Disease (RA ILD). The numbers within each section of the diagrams represent the count of unique or shared CSVs among the groups

Chromosomal variation may vary from patient to patient due to differences in genetic material and physiological conditions. To exclude individual differences, we intersected the chromosomal variants of patients in the same group. 1072, 1702, and 1117 genes were obtained in RA, RA LC and RA ILD, respectively (Fig. 2).

### Determination of specific CSVs in each disease and related functional analysis

**Fig. 3:**
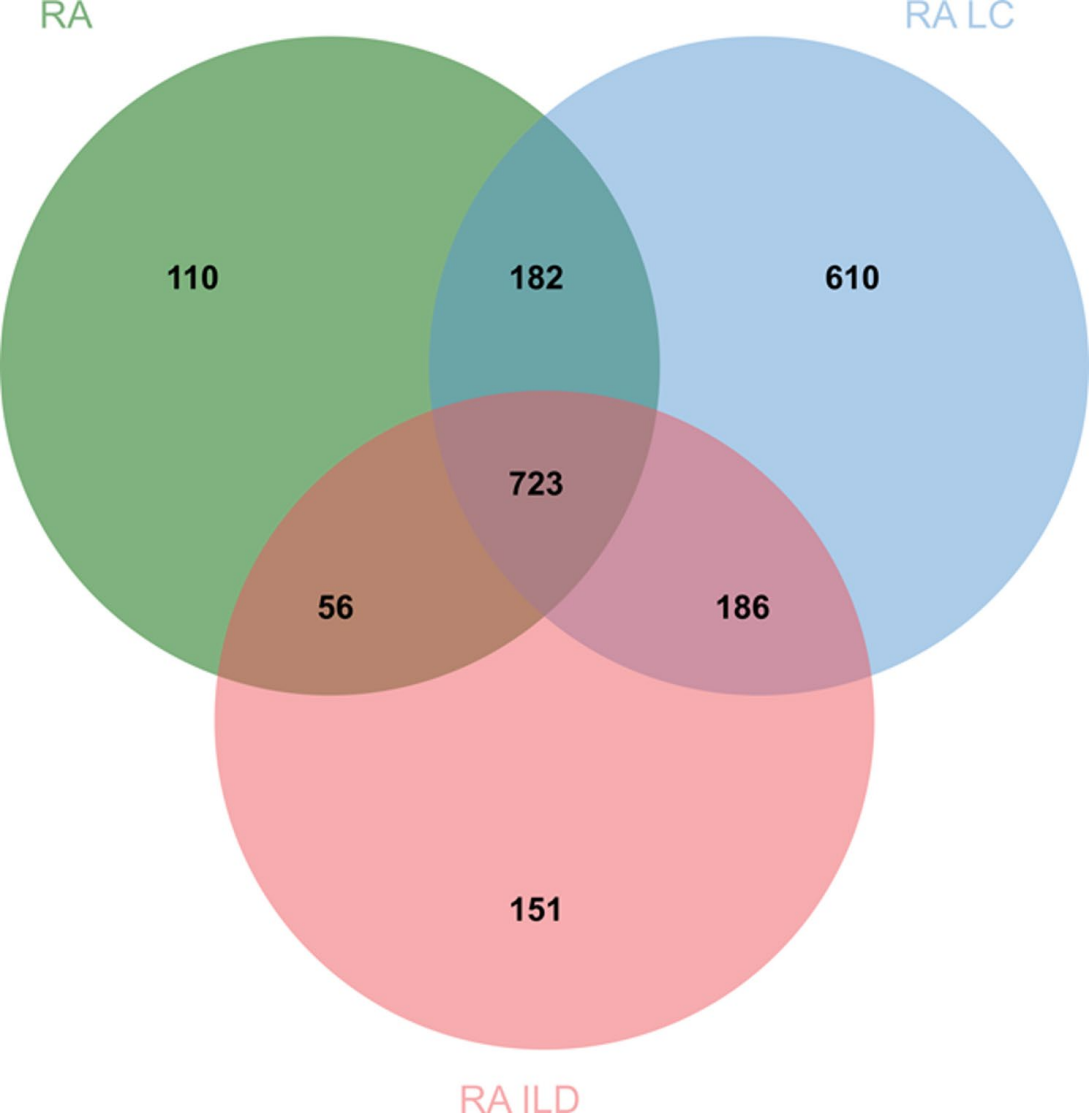
Key variant genes in CSVs between the three diseases

After obtaining each group’s mutated genes, the differences between different diseases were subsequently analyzed. RA LC had the most specific variant genes (610). RA and RA ILD had 110 and 151 specific variant genes, respectively. The three diseases shared a total of 723 common variant genes (Fig. 3). Next, the specific genetic factors between different diseases were explored by analyzing and mining the functions of the above key genes.

**Fig. 4:**
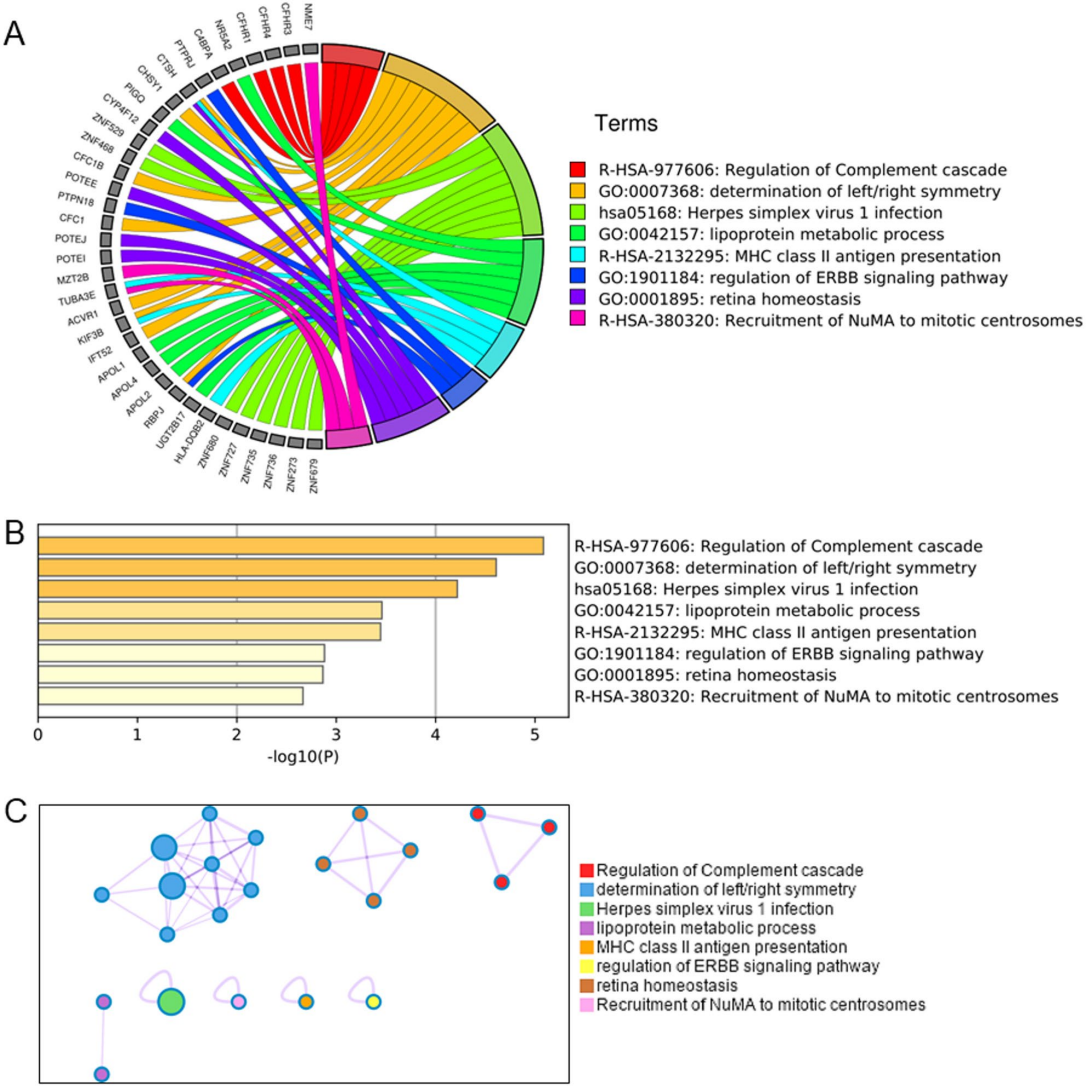
**A** Distribution of genes in RA CSVs. **B** Enrichment analysis of genes in RA-specific CSVs. **C** Network layout of genes in RA-specific CSVs

Previous analyses have found that each disease had some specific chromosomal variants, but their functions were unclear. We first identified the top 10 statistically enriched terms, including Gene Ontology (GO)/Kyoto Encyclopedia of Genes and Genomes (KEGG), canonical pathways, and hall mark gene sets. There were 110 specific variant genes of RA, and three terms related to immunity were obtained by functional analysis: Regulation of complement cascade, herpes simplex virus 1 infection and MHC class II antigen presentation. In addition, determination of left/right symmetry and lipoprotein metabolic process were also obtained (Fig. 4A). Regulation of Complement cascade, determination of left/right symmetry and herpes simplex virus 1 infection had the highest significance (Fig. 4B). We then selected a subset of representative terms from this cluster and converted them to a network layout (Fig. 4C). The Molecular Complex Detection (MCODE) analysis identified the top 3 components: determination of left/right symmetry, MHC class II antigen presentation and regulation of complement cascade.

**Fig. 5:**
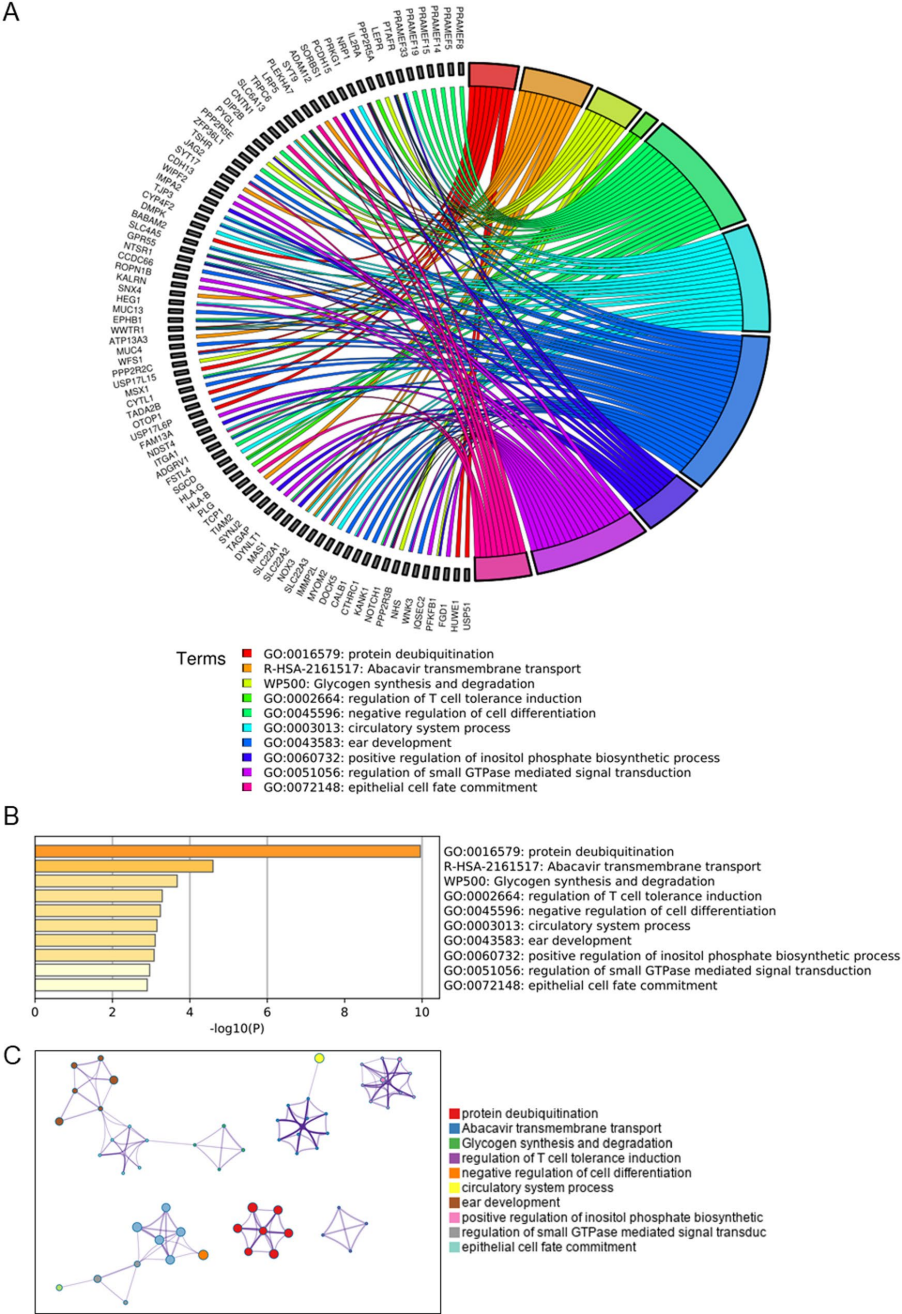
**A** Distribution of genes in RA LC-specific CSVs. **B** Enrichment analysis of genes in RA LC-specific CSVs. **C** Network layout of genes in RA LC-specific CSVs

RA LC has 610 specific variant genes, and enrichment analysis yields five key terms: negative regulation of cell differentiation, protein deubiquitination, abacavir transmembrane transport, glycogen synthesis & degradation and regulation of T cell tolerance induction et al. (Fig. 5A). The significance analysis showed that the term protein deubiquitination had the highest significance of –log10(P) = 10.0 (Fig. 5B). The Molecular Complex Detection (MCODE) analysis also identified protein deubiquitination as one of the essential components (Fig. 5C), which has not been reported in RA LC research.

**Fig. 6:**
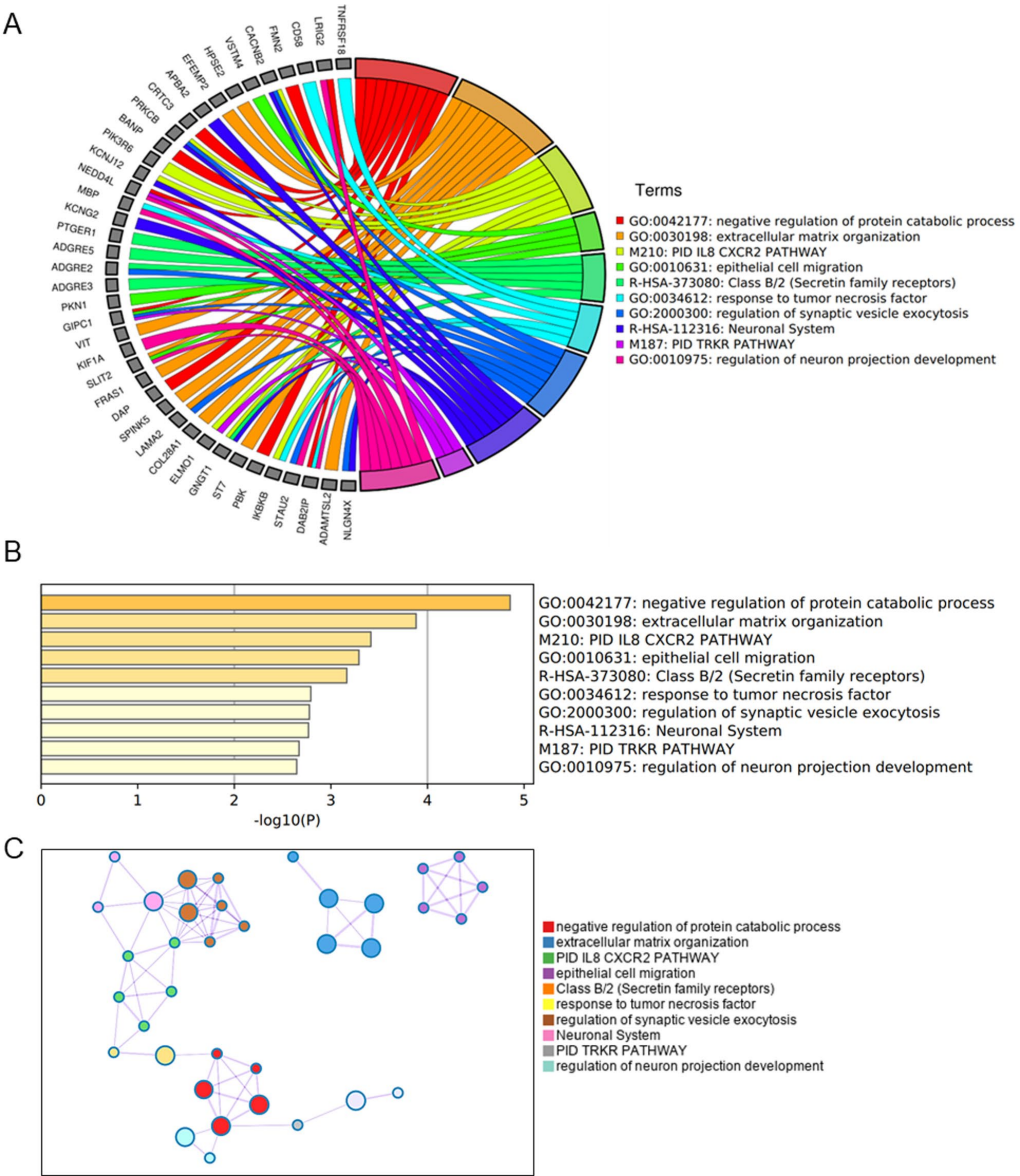
**A** Distribution of genes in RA ILD -specific CSVs. **B** Enrichment analysis of genes in RA ILD -specific CSVs. **C** Network layout of genes in RA ILD -specific CSVs

RA ILD has 151 specific variant genes, and enrichment analysis yields seven key terms: negative regulation of protein catabolic process, extracellular matrix organization, IL8 CXCR2 PATHWAY, epithelial cell migration, class B/2 (Secretin family receptors), response to tumor necrosis factor, regulation of synaptic vesicle exocytosis, neuronal system TRKR pathway and regulation of neuron projection development (Fig. 6A). The significance analysis showed that negative regulation of protein catabolic process, extracellular matrix organization, IL8 CXCR2 PATHWAY and epithelial cell migration had statistically significant differences (Fig. 6B). The Molecular Complex Detection (MCODE) analysis also identified the IL8 CXCR2 pathway as a hub for connecting neuronal system, regulation of synaptic vesicle exocytosis and response to tumor necrosis factor. The extracellular matrix organization and epithelial cell migration existed independently (Fig. 6C).

### Specific genetic regulatory network in different disease pathways

**Fig. 7:**
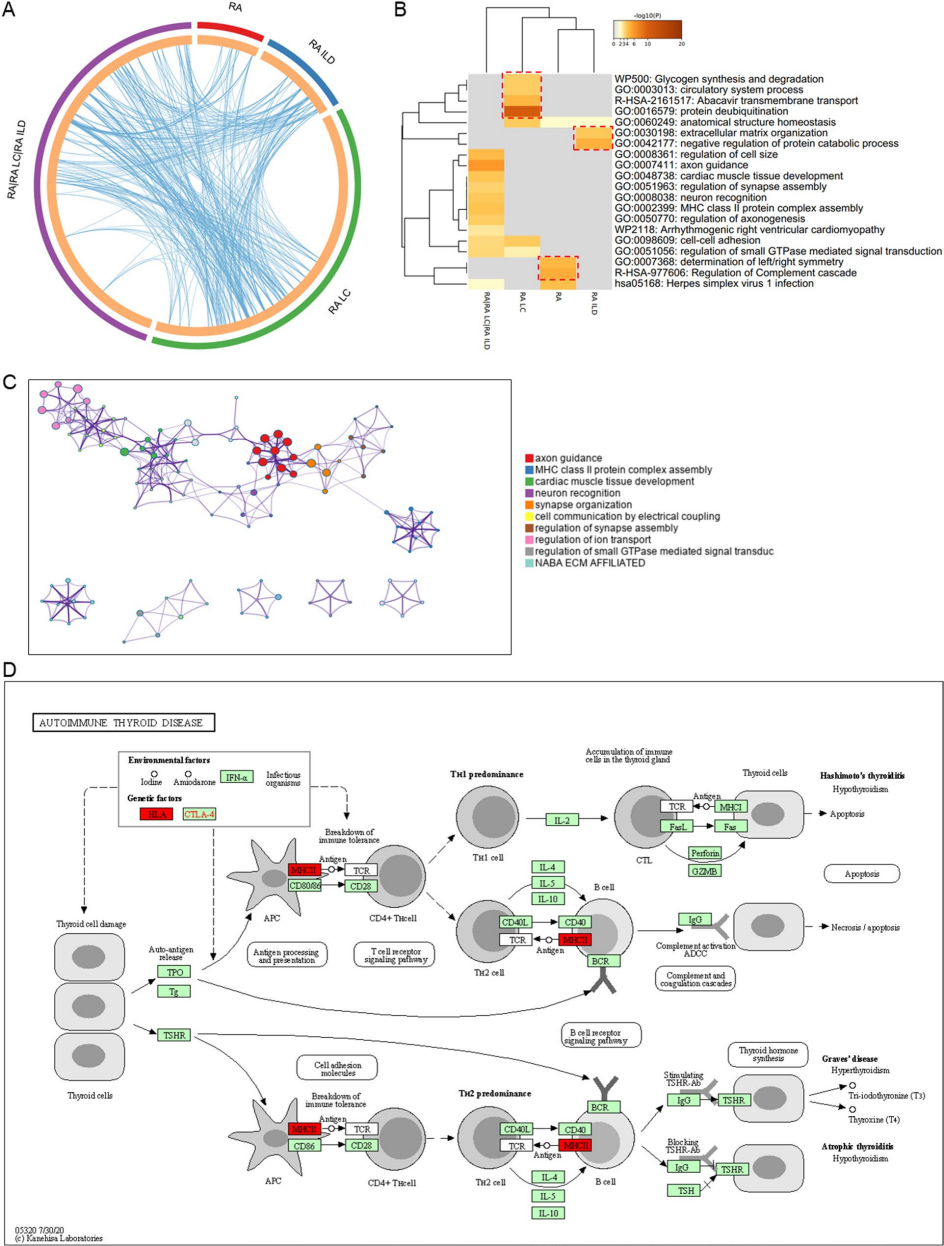
Comprehensive analysis of chromosomal variants in the three diseases. **A** The number of chromosomal variant genes and association analysis for each disease. **B** Common and specific pathways for the three diseases. Genes connected by the curves are in the same pathway. **C** Distribution of common variant genes across the three diseases in the autoimmune process. RA|RA LC|RA ILD: the intersection of the three diseases. **D** Key variant genes identified in MHC class II-mediated antigen presentation (in red)

All three diseases have certain pulmonary manifestations, and specific genetic feature plays a vital role. We performed an intergroup comparison to explore the relation and differences of key chromosomal variations between the diseases. Since RA is the basic disease in all groups, the common variant genes were the most (RA|RA LC|RA ILD). Then RA LC, RA ILD, and RA followed in order, with arcuate connections in the middle indicating the same pathways contain the genes (Fig. 7A). The more variant genes specific to RA LC suggest the more cancer-associated genetic factors, while ILD with the less. There are more links between RA LC and RA ILD than RA. The functional analysis of multiple groups showed that RA|RA LC|RA ILD enriched the most functional terms (Fig. 7B), with the highest significance for axon guidance. Protein-protein interaction analysis for common genes in RA|RA LC|RA ILD revealed axon guidance and cardiac muscle tissue development in the middle positions acting as hubs, while MHC class II protein complex assembly and regulation of ion transport were in the edge positions (Fig. 7C). MHC class II molecules present peptide fragments to T cells for immune recognition and thereby activate adaptive immunity in vivo, so MHC class II-restricted antigen presentation is critical for CD4^+^ T cell-dependent immune responses [32] (Fig. 7D). Regulation of Complement cascade and determination of left/right symmetry were enriched in RA. The complement system is an essential component of the innate immune system. It is essential for defense against microbial infections and for the clearance of immune complexes and damaged cells [33]. Complement has been implicated in the pathogenesis of RA; Elevated levels of complement activation products have been measured in the plasma, synovial fluid, and synovial tissue of patients. Complement polymorphisms are associated with RA in genome-wide association studies [34]. Activation of the complement system drives local inflammatory responses through metabolic reprogramming of synovial fibroblasts [35]; Synovial C3D levels were increased in active RA joints compared to controls [36]. These results suggest that complement activation plays an essential role in the induction and development of RA. Studies in recent years have demonstrated that anti-TNF agents are effective for the treatment of RA, and the reduction of complement activation may be one of the mechanisms by which TNFα inhibitors exert their effectiveness in inflammatory arthritis [37]. Therefore, inhibition of complement activation may become one of the targets for the treatment of RA. There have been some relevant studies on complement abnormalities; however, the association of determination of left/right symmetry and RA is still lacking. We annotated the locations of key mutated genes in the body’s immune pathways that are critical for antigen presentation.

**Fig. 8:**
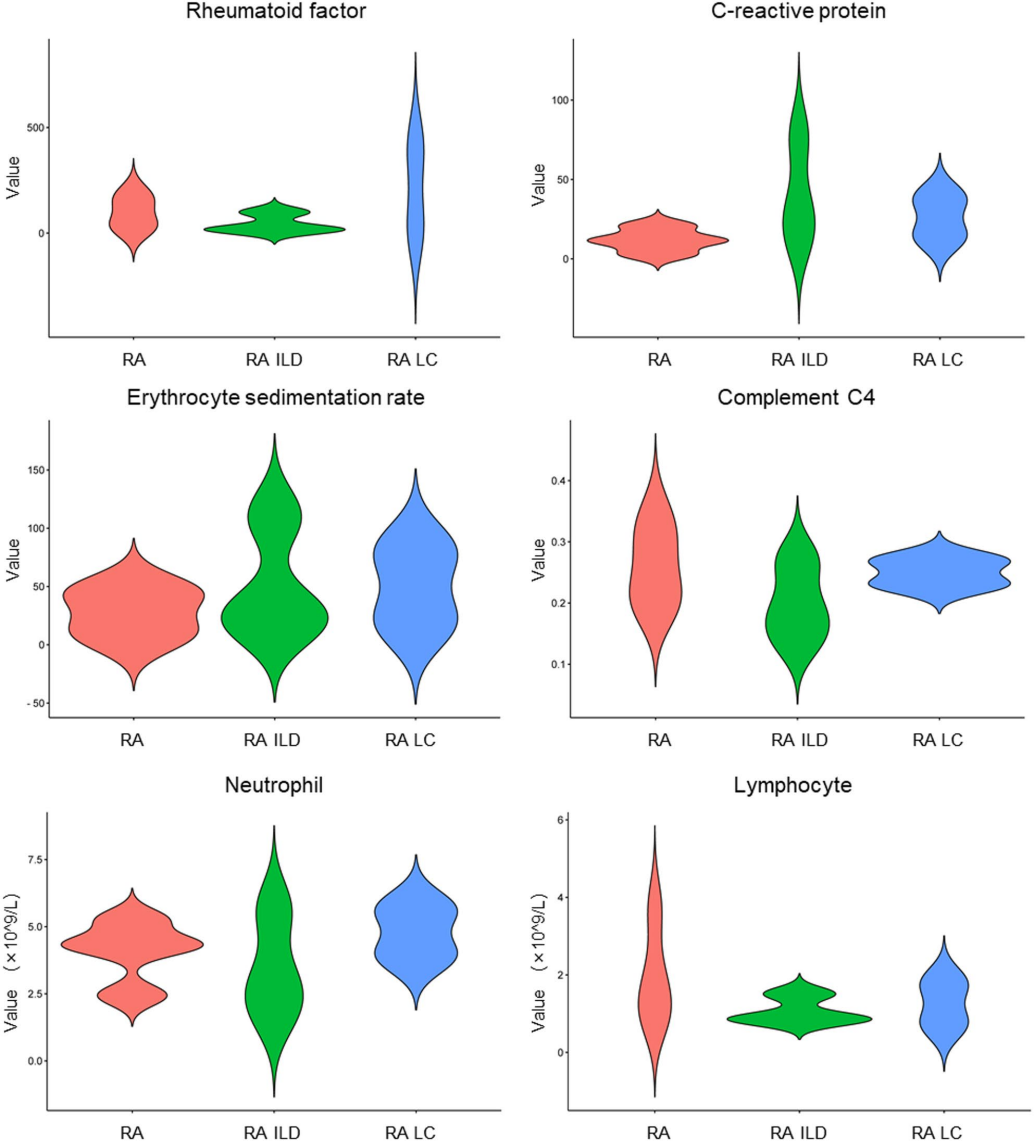
Hematological clinical data

The analysis of CSV highlighted the crucial role of MHC class II molecules in immune recognition and the complement system in the pathogenesis of RA. Building on this, we further analyzed the changes in various hematological biomarker levels across the three disease groups. Hematological clinical data revealed significant variations in biomarker levels, emphasizing the role of these immune components. Rheumatoid factors were elevated in RA and RA LC, with median values of 86.55 and 213.50, respectively, while RA ILD had a notably lower median value of 18.8. This elevation in rheumatoid factors suggests pronounced immune dysfunction, likely linked to dysregulated MHC class II-mediated antigen presentation. C-reactive protein (CRP) levels were significantly higher in RA ILD and RA LC, being three and two times greater, respectively, than in RA. All three disease groups had CRP levels exceeding the normal threshold of 5 mg/L, indicating heightened systemic inflammation and complement activation. The erythrocyte sedimentation rate (ESR) showed a similar pattern, being lower in RA compared to RA ILD and RA LC, with the latter two groups indicating more acute inflammatory processes. Additionally, the levels of neutrophils and lymphocytes were lower in RA ILD than in RA and RA LC, reflecting distinct inflammatory profiles, potentially driven by immune system dysregulation. These findings, combined with genetic regulatory network analysis, underscore the complex interplay between MHC class II molecules, the complement system, and disease-specific pathways in RA, RA LC, and RA ILD (Fig. 8).

## Discussion

This study aimed to identify differential CSVs among RA, RA LC and RA ILD. RA was chosen as the underlying disease because patients with RA are known to be at increased risk of malignancy [38–40]. Pulmonary manifestations are common in RA, and the genetic causes of lung disease in patients with RA were explored in this study.

While meta-analyses suggest that patients with RA have an increased risk of lung cancer compared with the general population [41, 42], the role of genetic factors is not clear. Therefore, we focused on the functions of disease-associated chromosomal variants encompassing genes and found some variations and associations between different diseases.

We observed that protein deubiquitination was absent in RA LC. Deubiquitinating enzymes are a large class of proteases, and changes in deubiquitinating enzymes have been linked to tumor cell proliferation and survival. For example, the downregulation of deubiquitinating enzyme USP12 promotes mouse and human lung tumor growth and promotes an immunosuppressive microenvironment [43]. Compared with healthy controls, the genes specific to RA are enriched in axon guidance [44]. Abnormal levels of Netrin 1, an axon guidance factor, had been detected in the synovial fluid of patients with RA. Netrin 1 has been proven to be a key regulator of osteoclast differentiation. Although the related mechanism in osteoclasts is mainly unknown, it could be a novel therapeutic target for RA bone destruction [45, 46]. MHC class II antigen presentation is strongly associated with immune function. Antigenic peptide-loaded MHC class II molecules are constitutively expressed on the surface of professional antigen-presenting cells (APCs), including dendritic cells, B cells, macrophages, and thymic epithelial cells [47]. The observed elevations in rheumatoid factor in RA LC, suggest that MHC class II molecules play a critical role in the immune response.

Interestingly, the loss of extracellular matrix organization was significantly enriched in RA ILD. The lung extracellular matrix plays a vital role in the normal structure of the lung [48], and its properties are essential in response to changes in lung disease. However, they are poorly understood. Here we found null mutations associated with the extracellular matrix organization in interstitial lung disease, including proteins VIT, ST7, ADAMTSL2, SPINK5, EFEMP2, HPSE2, COL28A1, LAMA2, SLIT2 and FRAS1.

A limitation of this study is the small sample size, which limits the statistical significance of the comparisons. The incidence of RA is 0.5–1.0.5.0% [3–5], and the average incidence of lung cancer was 0.059% [49], which resulted in fewer patients suffering from both diseases. Another limitation is sex predilection, with RA more common in women and lung cancer more common in men. As RA is the basic disease of this study, most of the patients are women (89%), and male patients are insufficient.

## Conclusion

Determining the genetic factors of RA, LC and ILD is a significant challenge that plays a vital role in detecting and treating diseases. Based on the comprehensive analysis of chromosome variation, we described the genetic characteristics of RA compared with related lung diseases, and revealed the related hematological characteristics. Identifying genetic factors in defined disease groups can not only explain the limitations of current pathogenesis but also provide insights for discovering promising targets and pathways.

## Supplementary Information

Supplementary Material 1.

## Data Availability

All data generated or analysed during this study are included in this published article. All chromosome structural variation data are in the supplementary files.

## References

[1] Seror R, Fautrel B, Lafourcade A, De-Rycke Y, Mariette X, Tubach F. Op0124 Risk Of Malignancies Across Biologic Classes In Rheumatoid Arthritis: Analysis Of A National Claim Database. Ann Rheum Dis. 2020;79:81-82.

[2] Zhang L, Qiliang Z, Yuan F. Liu Min: Lung cancer in patients with and without rheumatoid arthritis: A propensity score-matched survival analysis cohort study. Thorac Cancer. 2020;11(6):1406-13. 10.1111/1759-7714.13388 PMC7262940 32220060

[3] Aletaha D, Smolen JS. Diagnosis and management of rheumatoid arthritis: a review. JAMA. 2018;320(13):1360–72. 30285183 10.1001/jama.2018.13103

[4] Sparks JA. Rheumatoid arthritis. Ann Intern Med. 2019;170(1):Itc1–16. 30596879 10.7326/AITC201901010

[5] Silman Alan J, Pearson Jacqueline E. Epidemiology and genetics of rheumatoid arthritis. Arthritis Res. 2002;4(Suppl 3):S265–72. 12110146 10.1186/ar578 PMC3240153

[6] Lee DM, Weinblatt ME. Rheumatoid arthritis. Lancet (London England). 2001;358(9285):903–11. 11567728 10.1016/S0140-6736(01)06075-5

[7] Scott DL, Wolfe F, Huizinga TW. Rheumatoid arthritis. Lancet (London England). 2010;376(9746):1094–108. 20870100 10.1016/S0140-6736(10)60826-4

[8] Lopez-Olivo MA, Volk R, Krause KJ, Suarez-Almazor M. Ab0253 A review of smoking cessation strategies and lung cancer screening practices in patients with rheumatoid arthritis. Ann Rheum Dis. 2020;79:1426.

[9] Curtis JR, Lee EB, Kaplan IV, Kwok K, Geier J, Benda B, Soma K, Wang L, Riese R. Tofacitinib, an oral Janus kinase inhibitor: analysis of malignancies across the rheumatoid arthritis clinical development programme. Ann Rheum Dis. 2016;75(5):831–41. 25902789 10.1136/annrheumdis-2014-205847 PMC4853586

[10] Chatzidionysiou K, di Giuseppe D, Soderling J, Catrina A, Askling J. Risk of lung cancer in rheumatoid arthritis and in relation to autoantibody positivity and smoking. RMD open. 2022;8(2):e002465. 10.1136/rmdopen-2022-002465 PMC9594582 36270743

[11] McInnes IB, Schett G. The pathogenesis of rheumatoid arthritis. N Engl J Med. 2011;365(23):2205–19. 22150039 10.1056/NEJMra1004965

[12] Yarwood A, Huizinga TW, Worthington J. The genetics of rheumatoid arthritis: risk and protection in different stages of the evolution of RA. Rheumatology (Oxford). 2016;55(2):199–209. 25239882 10.1093/rheumatology/keu323 PMC4710800

[13] Dedmon LE. The genetics of rheumatoid arthritis. Rheumatology (Oxford, England). 2020;59(10):2661–70. 32638005 10.1093/rheumatology/keaa232

[14] Suzuki A, Terao C, Yamamoto K. Linking of genetic risk variants to disease-specific gene expression via multi-omics studies in rheumatoid arthritis. Semin Arthritis Rheum. 2019;49(3S):S49–53. 31779853 10.1016/j.semarthrit.2019.09.007

[15] Bronson PG, Criswell LA, Barcellos LF. The MHC2TA -168A/G polymorphism and risk for rheumatoid arthritis: a meta-analysis of 6861 patients and 9270 controls reveals no evidence for association. Ann Rheum Dis. 2008;67(7):933–6. 17875550 10.1136/ard.2007.077099 PMC2951320

[16] Julià A, Blanco F, Fernández-Gutierrez B, González A, Cañete JD, Maymó J, et al. Identification of IRX1 as a risk locus for rheumatoid factor positivity in rheumatoid arthritis in a genome-wide association study. Arthritis Rheum. 2016;68(6):1384–91. 10.1002/art.39591 26815016

[17] Viatte S, Plant D, Han B, Fu B, Yarwood A, Thomson W, et al. Association of HLA-DRB1 haplotypes with rheumatoid arthritis severity, mortality, and treatment response. JAMA. 2015;313(16):1645–56. 25919528 10.1001/jama.2015.3435 PMC4928097

[18] Okada Y, Eyre S, Suzuki A, Kochi Y, Yamamoto K. Genetics of rheumatoid arthritis: 2018 status. Ann Rheum Dis. 2019;78(4):446–53. 30530827 10.1136/annrheumdis-2018-213678

[19] Okada Y, Suzuki A, Ikari K, Terao C, Kochi Y, Ohmura K, et al. Contribution of a non-classical HLA gene, HLA-DOA, to the risk of rheumatoid arthritis. Am J Hum Genet. 2016;99(2):366–74. 27486778 10.1016/j.ajhg.2016.06.019 PMC4974094

[20] Begovich AB, Carlton VE, Honigberg LA, Schrodi SJ, Chokkalingam AP, Alexander HC, et al. A missense single-nucleotide polymorphism in a gene encoding a protein tyrosine phosphatase (PTPN22) is associated with rheumatoid arthritis. Am J Hum Genet. 2004;75(2):330–7. 15208781 10.1086/422827 PMC1216068

[21] Gregersen PK. Pathways to gene identification in rheumatoid arthritis: PTPN22 and beyond. Immunol Rev. 2005;204:74–86. 15790351 10.1111/j.0105-2896.2005.00243.x

[22] Danoy P, Wei M, Johanna H, Jiang L, He D, Sun L, Zeng X, Visscher PM, Brown MA, Xu H. Association of variants in MMEL1 and CTLA4 with rheumatoid arthritis in the Han Chinese population. Ann Rheum Dis. 2011;70(10):1793–7. 21784728 10.1136/ard.2010.144576

[23] Jiang L, Yin J, Ye L, Yang J, Hemani G, Liu AJ, et al. Novel risk loci for rheumatoid arthritis in Han Chinese and congruence with risk variants in Europeans. Arthritis Rheumatol. 2014;66(5):1121–32. 24782177 10.1002/art.38353

[24] Okada Y, Wu D, Trynka G, Raj T, Terao C, Ikari K, Kochi Y, Ohmura K, Suzuki A, Yoshida S, et al. Genetics of rheumatoid arthritis contributes to biology and drug discovery. Nature. 2014;506(7488):376–81. 24390342 10.1038/nature12873 PMC3944098

[25] Freudenberg J, Gregersen P, Li W. Enrichment of genetic variants for rheumatoid arthritis within T-cell and NK-cell enhancer regions. Mol Med (Cambridge Mass). 2015;21:180–4. 25794145 10.2119/molmed.2014.00252 PMC4503658

[26] Barton A, Thomson W, Ke X, Eyre S, Hinks A, Bowes J, Plant D, Gibbons LJ, Consortium. YEAR. Rheumatoid arthritis susceptibility loci at chromosomes 10p15, 12q13 and 22q13. Nat Genet. 2008;40(10):1156–9. 18794857 10.1038/ng.218 PMC2662493

[27] Plenge RM, Seielstad M, Padyukov L, Lee AT, Remmers EF, Ding B, et al. TRAF1-C5 as a risk locus for rheumatoid arthritis–a genomewide study. N Engl J Med. 2007;357(12):1199–209. 17804836 10.1056/NEJMoa073491 PMC2636867

[28] Patsopoulos NA, Ioannidis JP. Susceptibility variants for rheumatoid arthritis in the TRAF1-C5 and 6q23 loci: a meta-analysis. Ann Rheum Dis. 2010;69(3):561–6. 19401279 10.1136/ard.2009.109447

[29] Remmers EF, Plenge RM, Lee AT, Graham RR, Hom G, Behrens TW, de Bakker PI, Le JM, Lee HS, Batliwalla F, et al. STAT4 and the risk of rheumatoid arthritis and systemic lupus erythematosus. N Engl J Med. 2007;357(10):977–86. 17804842 10.1056/NEJMoa073003 PMC2630215

[30] Huang daW, Sherman BT, Lempicki RA. Systematic and integrative analysis of large gene lists using DAVID bioinformatics resources. Nat Protoc. 2009;4(1):44–57. 19131956 10.1038/nprot.2008.211

[31] Kanehisa M, Furumichi M, Sato Y, Ishiguro-Watanabe M, Tanabe M. KEGG: integrating viruses and cellular organisms. Nucleic Acids Res. 2021;49(D1):D545–51. 33125081 10.1093/nar/gkaa970 PMC7779016

[32] van Kasteren SI, Overkleeft H, Ovaa H, Neefjes J. Chemical biology of antigen presentation by MHC molecules. Curr Opin Immunol. 2014;26:21–31. 24556397 10.1016/j.coi.2013.10.005

[33] Noris M, Remuzzi G. Overview of complement activation and regulation. Semin Nephrol. 2013;33(6):479–92. 24161035 10.1016/j.semnephrol.2013.08.001 PMC3820029

[34] Giles JL, Choy E, van den Berg C, Morgan BP, Harris CL. Functional analysis of a complement polymorphism (rs17611) associated with rheumatoid arthritis. J Immunol. 2015;194(7):3029–34. 25725109 10.4049/jimmunol.1402956 PMC4367161

[35] Friščić J, Böttcher M, Reinwald C, Bruns H, Wirth B, Popp SJ, Walker KI, Ackermann JA, Chen X, Turner J, et al. The complement system drives local inflammatory tissue priming by metabolic reprogramming of synovial fibroblasts. Immunity. 2021;54(5):1002–e10211010. 33761330 10.1016/j.immuni.2021.03.003

[36] Doherty M, Richards N, Hornby J, Powell R. Relation between synovial fluid C3 degradation products and local joint inflammation in rheumatoid arthritis, osteoarthritis, and crystal associated arthropathy. Ann Rheum Dis. 1988;47(3):190–7. 2833185 10.1136/ard.47.3.190 PMC1003482

[37] Ballanti E, Perricone C, Muzio Gdi, Kroegler B, Chimenti MS, Graceffa D, Perricone R. Role of the complement system in rheumatoid arthritis and psoriatic arthritis: relationship with anti-TNF inhibitors. Autoimmun Rev. 2011;10(10):617–23. 21549221 10.1016/j.autrev.2011.04.012

[38] Mercer LK, Davies R, Galloway JB, Low A, Lunt M, Dixon WG, et al. Risk of cancer in patients receiving non-biologic disease-modifying therapy for rheumatoid arthritis compared with the UK general population. Rheumatology (Oxford, England). 2013;52(1):91–8. 23238979 10.1093/rheumatology/kes350 PMC3521445

[39] Hellgren K, Smedby KE, Feltelius N, Baecklund E, Askling J. Do rheumatoid arthritis and lymphoma share risk factors? A comparison of lymphoma and cancer risks before and after diagnosis of rheumatoid arthritis. Arthritis Rheum. 2010;62(5):1252–8. 20155827 10.1002/art.27402

[40] Dreyer L, Mellemkjær L, Andersen AR, Bennett P, Poulsen UE, Juulsgaard Ellingsen T, et al. Incidences of overall and site specific cancers in TNFα inhibitor treated patients with rheumatoid arthritis and other arthritides - a follow-up study from the DANBIO registry. Ann Rheum Dis. 2013;72(1):79–82. 22945500 10.1136/annrheumdis-2012-201969

[41] Simon Teresa A, Adam T, Gandhi Kunal K, Hochberg Marc C, Samy S. Incidence of malignancy in adult patients with rheumatoid arthritis: a meta-analysis. Arthritis Res Ther. 2015;17(1):212–212. 26271620 10.1186/s13075-015-0728-9 PMC4536786

[42] Smitten AL, Simon TA, Hochberg MC, Suissa S. A meta-analysis of the incidence of malignancy in adult patients with rheumatoid arthritis. Arthritis Res Ther. 2008;10(2):R45. 18433475 10.1186/ar2404 PMC2453765

[43] Yang Z, Xu G, Wang B, Liu Y, Zhang L, Jing T, et al. USP12 downregulation orchestrates a protumourigenic microenvironment and enhances lung tumour resistance to PD-1 blockade. Nat Commun. 2021;12(1):4852. 34381028 10.1038/s41467-021-25032-5 PMC8357983

[44] Hao R, Du H, Guo L, Tian F, An N, Yang T, Wang C, Wang B, Zhou Z. Identification of dysregulated genes in rheumatoid arthritis based on bioinformatics analysis. PeerJ. 2017;5:e3078. 28316886 10.7717/peerj.3078 PMC5356478

[45] Maruyama K, Kawasaki T, Hamaguchi M, Hashimoto M, Furu M, Ito H, et al. Bone-protective functions of Netrin 1 protein. J Biol Chem. 2016;291(46):23854–68. 27681594 10.1074/jbc.M116.738518 PMC5104911

[46] Mediero A, Ramkhelawon B, Perez-Aso M, Moore KJ, Cronstein BN. Netrin-1 is a critical autocrine/paracrine factor for osteoclast differentiation. J Bone Miner Res. 2015;30(5):837–54. 25483983 10.1002/jbmr.2421 PMC4689304

[47] Roche PA, Furuta K. The ins and outs of MHC class II-mediated antigen processing and presentation. Nat Rev Immunol. 2015;15(4):203–16. 25720354 10.1038/nri3818 PMC6314495

[48] Usman K, Hsieh A, Hackett TL. The role of MiRNAs in extracellular matrix repair and chronic fibrotic lung diseases. Cells. 2021;10(7):1706. 10.3390/cells10071706 PMC8304879 34359876

[49] Lu T, Yang X, Huang Y, Zhao M, Li M, Ma K, et al. Trends in the incidence, treatment, and survival of patients with lung cancer in the last four decades. Cancer Manag Res. 2019;11:943–53. 30718965 10.2147/CMAR.S187317 PMC6345192

